# Investor attention and corporate social responsibility of family businesses in Vietnam: The moderating role of CEO overpower

**DOI:** 10.1371/journal.pone.0306989

**Published:** 2024-07-19

**Authors:** Khoa Dang Duong, Hanh Thi Hong Nguyen, Phuc Huu Truong, Hoa Thanh Phan Le

**Affiliations:** 1 Faculty of Finance and Banking, Ton Duc Thang University, Ho Chi Minh City, Vietnam; 2 Faculty of Business Administration, Ton Duc Thang University, Ho Chi Minh City, Vietnam; 3 School of Finance and International Business, Saxion University of Applied Sciences, Deventer, Netherlands; 4 Faculty of Accounting and Auditing, Van Lang University, Ho Chi Minh City, Vietnam; University of Baltistan, PAKISTAN

## Abstract

This study examines the influence of investor attention and Chief Executive Officers (CEOs) power on Corporate Social Responsibility (CSR) within Vietnamese family businesses. Unlike most of the past literature, this study further investigates the potential moderating effects of CEOs’ power on the relationship between investor attention and CSR. Utilizing the dynamic system Generalized Method of Moments (GMM), this study analyzes a dataset comprising 116 Vietnamese family businesses from 2005 to 2020. The findings reveal an inverted U-shape between CEO power and CSR within family businesses; meanwhile, investor attention demonstrates a negative impact on CSR. Moreover, the results report that CEO power is a moderating factor in the relationship between investor attention and CSR. These results are consistent with various theoretical frameworks, including agency theory, overinvestment, career concern, career horizon, and conflict-resolution hypotheses. Finally, our study offers management implications to foster the sustainable development of CSR within family businesses, particularly within emerging markets.

## 1. Introduction

In the contemporary business landscape, corporate social responsibility (CSR) has become an integral component of corporate governance, signifying an expansion of companies’ endeavors to fortify governance effectiveness by embracing ethical business practices that promote accountability and transparency, thus ensuring enduring sustainability. Regarding governance, chief executive officers (CEOs) and investors are pivotal in shaping the vision and driving action within CSR initiatives. In that context, optimizing resource allocation towards CSR investments encounters challenges influenced by various theoretical frameworks, including agency theory, the overinvestment hypothesis, the career concern hypothesis, the career horizon hypothesis, the stakeholder theory, and the conflict-resolution hypothesis.

Vietnam is a developing country that depends heavily on fossil fuels for economic growth; therefore, environmental concerns have emerged as a critical topic. The Vietnamese government has implemented effective environmental laws and policies to encourage companies to adopt CSR and disclose their environmental impact responsibly. Furthermore, with the technical support of the German Confederation of Labor, the Vietnam General Confederation of Labor has implemented projects to raise awareness of CSR for trade union officials at all levels, including trade union officials of enterprises. Moreover, the CSR Award is an annual award announced in Vietnam in 2005. It has been awarded to hundreds of participating enterprises from all industries. In CSR research, most attention has been directed toward advanced economies like the United States (US) and the United Kingdom (UK). However, empirical studies on frontier markets like Vietnam still need to be more extensive, primarily due to data limitations. Additionally, CSR practices in different markets differ regarding institutions and governance [1], such as in developed and emerging markets. As a result, there is a research gap in the current literature about CSR in frontier markets. Hence, this study investigates corporate social responsibility (CSR) endeavors within Vietnam, illustrating its role as an emblematic frontier market in Asia.

According to the Family Business Survey 2021—Vietnam Report by PricewaterhouseCoopers (PwC), family businesses are vital to many economies worldwide. In Vietnam, the top 100 family businesses represent 25% of the GDP, and 33% of family business managers expect growth opportunities in 2022, surpassing other Asian countries [2]. In addition, family businesses have a unique ownership structure and governance, which can influence how CSR is implemented. For instance, family firms tend to have a longer-term focus, which can lead to a greater emphasis on social and environmental responsibility; however, they may also face challenges in implementing CSR due to conflicts of interest between investors and family members and a lack of formal governance structures. Therefore, examining how family businesses implement CSR in Vietnam is worthwhile.

Prior theories report mixed findings between CEO overpower and CSR investments. Besides, the agency theory and overinvestment hypothesis argue that powerful CEOs might engage in excessive CSR investments to bolster their reputation [3]. The career concern and career horizon hypotheses propose that CEOs prioritize CSR initiatives during their early tenure to decrease the likelihood of turnover, and they are no longer interested in CSR when they are about to retire. CSR benefits the CEO in the later career stages by improving long-term performance [4]. CEOs with short tenure have weak determinations to invest in CSR because they are less likely to accumulate the benefits of CSR engagement [5]. These theories and hypotheses suggest an inverted U-shape relationship between powerful CEOs and CSR engagement. In contrast, Sheikh [3] relies on the stakeholder theory to suggest a positive relationship between these variables. Powerful CEOs invest in CSR to benefit stakeholders and increase business value, not merely for personal reputation [3]. Therefore, there is an increase in the number of studies that found mixed results related to how powerful CEOs affect CSR, including Jiraporn and Chintrakarn [6], Li et al. [7], Sheikh [3], Oh et al. [5], Li et al. [8]. As prior studies report controversial findings and conflicts in theories, it is worthwhile to investigate how powerful CEOs affect CSR initiatives in Vietnam.

Existing theories have yet to conclude whether CEO power moderates the relationship between investor attention and CSR. According to agency theory and the over-investment hypothesis, it is suggested that excessive CEO influence may lead to an excessive allocation of resources toward CSR initiatives [3]. Furthermore, in line with the overinvestment hypothesis derived from agency theory, it proposes that managers may engage in disproportionate CSR spending when such actions yield personal benefits, such as enhancing their reputations as socially responsible leaders. Moreover, CEOs who possess greater control can make decisions that may disregard investor interests [9]. In contrast, Jo and Harjoto [10] propose that increased monitoring through diverse corporate governance mechanisms, such as oversight from institutional investors, is expected to decrease the motivation and opportunities for overconfident CEOs and insiders to engage in excessive CSR investments to enhance their reputations as socially responsible individuals or entities. Therefore, existing literature leaves a research gap in examining whether CEO power moderates the relationship between investor attention and the CSR engagement of family businesses in Vietnam.

This research aims to scrutinize the impact of CEO powers and investor attention on CSR endeavors. Unlike previous literature, this study also explores potential moderating effects of CEO power on the relationship between investor attention and CSR. To address the literature gap, we gathered data from 116 listed Vietnamese family firms, covering 2005 to 2020. In addition, this study is unique and complements the studies of Sheikh [3], Chen et al. [4], Oh et al. [5], Jiraporn and Chintrakarn [6], Li et al. [8], Jo and Harjoto [10], Xiang et al. [11], Arora and Dharwadkar [12] in the following manners. Firstly, they have yet to use the dynamic system Generalized Method of Moments (GMM) estimator, a reasonable method since it can overcome autocorrelation, heteroskedasticity, and endogeneity issues. This study utilized Durbin Watson, Wald, and Durbin-Wu-Hausman tests to confirm the presence of these violations and to ensure that the use of the GMM method is essential in this study. The results confirm that these violations exist. Therefore, following Duong et al. [13], Almustafa et al. [14], and Vo et al. [15], we employ a two-step GMM method to deal with all violations and ensure that the estimates obtained are reliable and efficient. This study also follows Duong et al. [16] to implement our robustness test across various subgroups of CEO power categorized by age.

This research presents remarkable findings. First, the results suggest an inverted U-shaped relationship between CEO power and CSR. This suggests that powerful CEOs positively impact firms’ CSR engagements. Nevertheless, if CEO power surpasses a certain threshold, CSR investments diminish due to this amplified power. Notably, in Vietnam, this turning point is estimated at 6.06. The findings are consistent with the agency theory, overinvestment hypothesis, career concern hypothesis, career horizon hypothesis, and studies of Chen et al. [4], Jiraporn and Chintrakarn [6]. Second, our results report that greater investor attention negatively affects CSR. The findings are consistent with the conflict-resolution hypothesis and the arguments of Arora and Dharwadkar [12]. Third, the study shows that powerful CEOs in companies with greater investor attention have a higher CSR engagement level than others, given that their companies have high investor attention. The findings align with the arguments put forth by Sheikh [3], Jo and Harjoto [10], agency theory, and the overinvestment hypothesis.

Additional analyses confirmed the robustness of our findings across CEO age subsamples. The results revealed the moderating role of investor attention and a consistent inverted U-shaped relationship between CEO power and CSR across CEO ages. However, when the CEO age exceeds 50, powerful CEOs in family businesses with investor attention negatively affect CSR engagements. High investor attention should reduce the incentive and opportunities of overconfident CEOs and insiders for CSR overinvestment due to goal conflicts relating to time horizons and uncertainty of outcomes [12].

Consequently, the current paper seeks to make the following contributions to the existing literature on corporate social responsibility in the following aspects. Firstly, this paper examines the interplay between investor attention, CEO power, and CSR, particularly focusing on the potential moderating role of CEO power in shaping the relationship between investor attention and CSR. Secondly, this study represents a pioneering endeavor to explore the impact of investor attention and CEO power on CSR initiatives within a transitional market in Asia. Such markets possess unique microstructures distinct from emerging and developed markets [16].

Thirdly, regarding variable measurement, most of the previous research employs individual proxy of CEO power, including Chen et al. [4], Oh et al. [5], Jiraporn and Chintrakarn [6], Li et al. [7], Park et al. [17]. Finkelstein [18] stated that power is a multifaceted concept, and to accurately measure it, we should consider an index that accounts for different power sources. Thus, in line with the works of Altunbaş et al. [19], Brodmann et al. [20], and Duong et al. [16], the principle component analysis (PCA) approach was used to construct the CEO power index in our research to reduce the risk of data overload.

Fourthly, to the best of our knowledge, this is one of the first studies examining how investor attention affects CSR. Previous papers only stop at the level of arguments or give results that indirectly show the relationship between them, including Arora and Dharwadkar [12], Radu and Smaili [21], and Bhuiyan and Nguyen [22]. Additionally, regarding variable measurement, we employed the method introduced by Da et al. [23] to quantify investor attention. We utilized the search frequency in the Google Search Volume Index (SVI), considered a direct and unambiguous measure of attention. The search volume provided by Google is considered a trustworthy representation of the general public attention. Furthermore, searching on Google indicates focused attention because individuals who look up a stock on the platform undoubtedly prioritize giving it their attention [23]. Moreover, SVI can provide information quickly and continuously, while regular surveys require time and effort to collect and analyze data. SVI allows researchers and investors to periodically and dynamically monitor and analyze attention levels. Finally, building upon the results obtained, this study aims to make significant contributions to both economic research and policy implications.

The paper is organized as follows: Section 2 provides an extensive literature review, Section 3 delineates the data and methodology utilized, Section 4 presents the findings, and Section 5 focuses on the ensuing discussion. Lastly, Section 6 concludes the research endeavor by offering implications.

## 2. Literature review

### 2.1. Theories

#### 2.1.1. Agency theory

Conflicts between the CEO and the firm concerning strategic decisions can hinder the firm’s ability to achieve good performance, with CSR being one aspect of such performance [24]. According to the agency theory, CSR can create an agency dilemma where managers invest in CSR initiatives to enhance personal standing. Insiders may invest excessively in CSR for reputation enhancement [25], and powerful CEOs can also leverage their influence to modify CSR investments for power consolidation [26]. In addition, according to Na et al. [9], powerful CEOs can exert influence over management decisions, potentially diminishing the board’s effectiveness. CEOs with greater control can make decisions disregarding investor interests [9]. Therefore, agency theory proposes that CEO overpower can result in overinvestment in CSR [3]. Moreover, according to the overinvestment hypothesis (based on agency theory), Jo and Harjoto [10] posit that managers might have a propensity to engage in excessive CSR investments when such endeavors confer personal advantages, such as augmenting their reputations as socially responsible individuals.

#### 2.1.2. Career concern hypothesis

On the other hand, as per the career concern hypothesis Chen et al. [4], CEOs prioritize CSR initiatives early in their tenure to alleviate career-related concerns. Good CSR performance reduces CEO turnover probability and is crucial for their evaluation. Moreover, CSR can generate long-term benefits for the company, which can benefit the CEO later in their career. Younger CEOs with longer career horizons display a higher propensity to actively engage in CSR investments as they can reap more significant benefits from such engagement [5]. According to this hypothesis, the effect of CEO overinvestment in CSR, as stated by the overinvestment hypothesis and agency theory, is enhanced when CEOs are in the early stages of their careers.

#### 2.1.3. Career horizon hypothesis

CEOs anticipating a short tenure, particularly those nearing retirement, might have less motivation to pursue CSR initiatives actively, even with comparable pressures from independent directors [4]. Oh et al. [5] posited that CEOs who approach or exceed the retirement age may be reluctant to make socially responsible decisions owing to the long-term return on investment associated with CSR initiatives. As a result, such CEOs are less likely to accrue the benefits of CSR engagement, reducing their motivation to engage in socially responsible decision-making. In addition, if executives reach their retirement age or the expiration of their agreement, it is documented as being unrelated to the company’s performance [27]. Therefore, according to the career horizon hypothesis, the effect of overinvestment in CSR decreases when CEOs reach a certain point in their tenure or age.

#### 2.1.4. Stakeholder theory

Enterprises exist within intricate natural and social connections, including shareholders, managers, employees, customers, local communities, investors, and networks. They encompass diverse actors from public and voluntary sectors, all falling under the stakeholder umbrella as groups or individuals capable of influencing or being influenced by the organization’s goals [28]. According to Sheikh [3], the stakeholder theory posits that CEOs engage in CSR activities to increase the firm value and balance stakeholders’ interests rather than seek personal reputation enhancement or private benefits. Stakeholder theory also holds that the CEO’s self-interest impact due to the agency theory, as discussed previously, would eventually decrease as the CEO’s ownership in the firm rises. In addition, a corporation ought to deliver value to all its stakeholders, encompassing investors, consumers, employees, local communities, and resources [29]. Hence, managers participate in CSR to fulfill their moral, ethical, and social obligations to stakeholders [10]. CSR engagements are considered a way to reduce stakeholder pressure and oversight Duong et al. [16]. Therefore, powerful CEOs with a more decisive influence on investment decisions may use CSR investment to balance stakeholders’ interests.

Regarding investor attention, firms are under pressure from their stakeholders, especially socially responsible investors [21], to comply with CSR [22]. Corporate social responsibility (CSR) disclosure emerges in response to societal pressure and heightened media attention [30]. Therefore, CSR actions send positive signals to investors, who then respond by investing in the company. Aguilera et al. [31] also argued that CSR strengthens relationships among core stakeholders. Therefore, stakeholder theory also suggests that investor attention leads to an increase in CSR.

#### 2.1.5. Conflict-resolution hypothesis

According to the conflict-resolution hypothesis, Arora and Dharwadkar [12] contend that managers could face pressure to scale back their involvement in CSR, particularly from institutional investors emphasizing short-term profits. This pressure stems from conflicting objectives regarding timeframes and the uncertainty surrounding CSR outcomes. In addition, Jo and Harjoto [10] suggest that increased oversight from various corporate governance mechanisms, such as institutional investor supervision, can mitigate the inclination and opportunities for overly confident CEOs and insiders to excessively invest in CSR activities solely to enhance their socially responsible image. If corporate governance mechanisms focus primarily on short-term gains, the expenses associated with CSR endeavors may surpass the benefits. Institutional investors who prioritize meeting short-term goals and performance metrics may not encourage managers to participate in CSR initiatives due to the unpredictable nature of their outcomes [12]. Consequently, the conflict-resolution hypothesis suggests that increasing investor attention may dampen CSR, particularly from institutions with short-term objectives.

### 2.2. CEO overpower and CSR

Li et al. [7] discovered a favorable relationship between CEO power and CSR disclosure in the United Kingdom. Following the agency theory, CEOs are urged to participate in CSR for opportunistic reasons such as strengthening their public image and marketing initiatives. It also reduces stakeholder pressure and oversight due to the stakeholder theory and conflict-resolution hypothesis or improves CEO compensation or tenure [16]. Additionally, Boone et al. [32] discovered that CEOs with self-centered attitudes find usefulness in delivering communal advantages only when they are compensated financially or socially. According to the agency theory and overinvestment hypothesis, powerful CEOs who prioritize their interests are primarily extrinsically motivated to invest in CSR when they expect direct financial gains from such endeavors.

**Hypothesis 1.** Powerful CEOs positively affect the CSR engagements of Vietnamese-listed family businesses.

Duong et al. [16] found that CEOs with greater power exhibit a negative association with CSR programs. This tendency towards decreased CSR investments can be attributed to the belief that such investments are superfluous and lead to a depletion of the firm’s free cash flow. Oh et al. [5] found that dimensions of CEO power, such as CEO age and tenure, negatively affect CSR. According to their assertion, CEOs who have reached or surpassed the retirement age are comparatively less inclined to accrue advantages from CSR involvement and, consequently, exhibit lower propensities to undertake socially responsible actions due to the career horizon hypothesis. In addition, with an increase in their tenure, CEOs are inclined to adhere to obsolete paradigms, which results in a decreased capacity for flexibility and adaptability. Consequently, their vigilance decreases toward the demands and anticipations of the firm’s stakeholders. This lack of attentiveness is negatively linked to the firm’s CSR. Moreover, Arora and Dharwadkar [12] also stated that short-term horizons on the part of managers would reduce CSR.

Based on Park et al. [17] findings, a noteworthy negative relationship exists between CEO ownership in the form of equity-based compensation (EBC) and CSR. The study suggests that CEOs who receive a higher proportion of their compensation in the form of equity are more likely to engage less in CSR and more inclined to take risks that can lead to higher financial returns for their company and shareholders due to stakeholder theory. Moreover, powerful CEOs are unlikely to launch CSR initiatives because they emphasize defending their positions and increasing shareholder value [16]. Li et al. [8] also discovered a negative association between CEO power and CSR.

**Hypothesis 2.** Powerful CEOs negatively affect the CSR engagements of Vietnamese-listed family businesses in Vietnam.

According to Jiraporn and Chintrakarn [6], CEO power and CSR involvement have an inverted U-shape connection. There is a favorable association between the allocation of resources to CSR and the power of a CEO. This implies that the CEO may derive some personal benefits from such investments due to agency theory and the overinvestment hypothesis. However, once the CEO reaches a certain level of power and perceives their position as secure, they may develop a negative outlook toward CSR and decrease their investment. Additionally, highly powerful CEOs may view CSR investments as hindering their ability to exploit free cash flow.

Concerning CEO tenure, Chen et al. [4] discovered that CSR performance reaches its highest point within the initial three years of a CEO’s tenure, after which it gradually declines as the CEO’s tenure progresses. They stated that CEOs are incentivized to prioritize CSR in the initial stages of their tenure as a strategy to alleviate potential career concern issues, given that CSR is a critical aspect of their performance evaluation and A CEO’s strong CSR performance during their early tenure is linked to a reduced probability of CEO turnover (career concern hypothesis). Furthermore, they stated that CEOs in the early stages of their tenure have more significant incentives to get involved in CSR than those in later phases. This is because CSR efforts can generate long-term benefits for the firms, ultimately benefiting the CEOs in the future. In terms of CEO age, Oh et al. [5] stated that CEOs nearing or surpassing retirement age are less likely to benefit from CSR engagement due to the extended payback period associated with CSR investments. As a result, they demonstrate a lesser desire to make socially responsible decisions according to the career horizon hypothesis. Conversely, younger CEOs, who possess relatively extended career horizons, are more inclined to engage in CSR investment as they receive greater rewards from such engagements. Indeed, businesses that actively participate in CSR often pay their CEOs higher salaries than those that do not engage in CSR activities [8].

**Hypothesis 3.** Powerful CEOs have an inverted U-shape impact on the CSR engagements of Vietnamese-listed family businesses.

### 2.3. Investor attention and CSR

Over the past decade, environmental, social, and governance (ESG) factors have gained significant popularity among investors, partly due to the influence of CSR and, more recently, discussions on corporate sustainability [33]. Therefore, stakeholders, such as investors, communities, or environmental groups, pressure firms to comply with CSR due to stakeholder theory [22]. Moreover, Radu and Smaili [21] stated that socially responsible investors who are increasingly interested in CSR might exert pressure on corporations to improve CSR performance, suggesting that increasing investor attention positively affects CSR. Jo and Harjoto [10] found that investor supervision, as an external corporate governance (CG) device, positively affects CSR. The conflict-resolution hypothesis suggests that managers can utilize CSR engagement to settle stakeholder disputes when multiple CG mechanisms interpret CSR as an effort to resolve conflicts among various stakeholders.

**Hypothesis 4.** Investor attention positively affects the CSR engagements of Vietnamese-listed family businesses.

Corporate social responsibility can enhance firm performance, but the outcomes are mainly uncertain and long-term. Consequently, stakeholders with a long-term perspective are more likely to view CSR as beneficial. However, if CG mechanisms emphasize short-term results, the costs associated with CSR may outweigh the benefits. Institutional investors prioritizing meeting short-term objectives and performance targets may not advocate for managers to engage in CSR initiatives, given that the outcomes are uncertain [12]. Therefore, we assume that an escalation in investor attention is anticipated to cause a decline in firms’ CSR performances due to institutional investors’ pressure to fulfill short-term financial objectives.

**Hypothesis 5.** Investor attention negatively affects the CSR engagements of Vietnamese-listed family businesses.

### 2.4. The potential moderating role of CEO power on the relationship between investor attention and CSR

Jo and Harjoto [10] indicate that increased monitoring through diverse corporate governance mechanisms, like institutional investor oversight, is likely to diminish the motivation and opportunities for overconfident CEOs and insiders to engage in CSR overinvestment as a means to bolster their reputations as socially responsible individuals or entities. Arora and Dharwadkar [12] also argued that managers might experience pressure to reduce their engagement in CSR, especially from institutional investors who prioritize short-term returns. This is because of goal conflicts relating to time horizons and uncertainty of outcomes. However, according to Na et al. [9], CEOs with greater control can make decisions that may disregard investor interests. Due to the over-investment hypothesis, insiders might invest more in CSR initiatives to enhance their reputation [25], while influential CEOs could use their power to shape CSR strategies for personal gain [26]. Additionally, Na et al. [9] suggest that powerful CEOs may influence managerial decisions, undermining the board’s effectiveness and disregarding investor interests or attention. As proposed by agency theory, this concentration of power in CEOs may lead to excessive investment in CSR [3]. Furthermore, in line with the overinvestment hypothesis derived from agency theory, Jo and Harjoto [10] suggest that managers may be inclined to overcommit resources to CSR activities when it benefits their reputation as socially responsible individuals.

**Hypothesis 6.** In listed family businesses, powerful CEOs drive increased CSR involvement, even amidst investor attention.

## 3. Data and methodology

### 3.1. Data

Data were gathered from 116 family businesses in Vietnam listed on the Ho Chi Minh Stock Exchange and Hanoi Stock Exchange from 2005 to 2020. In order to provide a suitably high sample size for regression analysis and to reduce biases, data collecting started in 2005. We collect family business data by hand from Vietstock and annual financial reports. We follow Tran et al. [2] to remove financial firms due to their substantial dependence on financial leverage, which might lead to abnormal excess leverage ratios in financial firms and signify distress risk in non-financial sectors. We also follow Duong et al. [16] to winsorize all variables at 5% and 95% to address concerns related to extreme values. Additionally, observations lacking sufficient data for calculating the required variables were removed from the dataset. To identify family businesses, we applied criteria based on the involvement of internal stakeholders who share surnames in the management and governance of the business. This methodology aligns with the approach utilized by Eckrich et al. [34] and Diéguez-Soto et al. [35]. For instance, when the chairman’s last name corresponds to the last names of managers or block holders, the enterprise is classified as a family business. The final data is an unbalanced sample of 985 annual observations from 2005 to 2020.

### 3.2. Variable definitions

#### 3.2.1. Corporate social responsibility

We utilize the disclosure technique proposed by Aggarwal and Singh [36] and Duong et al. [16] to quantify and categorize CSR disclosure into four areas: environment, society, employees, goods, consumers, and suppliers. The CSR disclosure index ranges from 0 to 4, reflecting the level of CSR disclosure. In Vietnam, the lack of a social rating agency makes the disclosure approach using annual report content analysis more preferable, unveiling the complexity of CSR practices [16].

#### 3.2.2. CEO overpowers

Following Duong et al. [16], Altunbaş et al. [19], and Brodmann et al. [20], we adopt the principle component analysis (PCA) approach to estimate the CEO power index. This method reduces the risk of overload by transforming high-dimensional variables, such as CEO age, CEO tenure, and CEO ownership, into a single low-dimensional index, effectively capturing CEO power. The CEO’s authority is represented by CEO ownership, and longer CEO tenure boosts CEO power [3, 16]. Liu and Jiraporn [37] argued that older CEOs nearing retirement might reduce their involvement as they prepare to pass the company’s leadership to their successors. Moreover, these three dimensions of CEO power have been widely used by prior studies such as Chen et al. [4], Oh et al. [5], and Li et al. [8] to estimate the relationship between CEO power and CSR. The results of PCA for CEO power are shown in S1 Appendix.

#### 3.2.3. Investor attention

Our study employs the approach introduced by Da et al. [23] to assess investor interest through the utilization of search frequency in the Google Search Volume Index (SVI). They suggested that Google’s search volume is a dependable indicator of the general public’s Internet search behavior. Furthermore, they argued that searching for a stock on Google signifies concentrated attention, as individuals actively seek information about that specific stock. Consequently, the aggregate search frequency on Google presents a direct and lucid measure of the level of attention investors direct toward a particular stock [23].

Notably, to account for systematic differences across industries as well as business cycle influences, we follow Chauhan [38], Cui and Leung [39] to adjust CEO power and investor attention for industry and business cycle variations by subtracting the median CEO power and investor attention by industry and year from each CEO power and investor attention. The discrepancy is then adjusted by industry and year standard deviations to account for variations in variance.

### 3.3. Other control variables

#### 3.3.1. Leverage

We follow Chen et al. [4] in controlling for leverage (LEV) ratio, which is determined by dividing total liabilities by total assets. Their findings point to a beneficial association between leverage and CSR. Firms with idle cash resources are more able to afford CSR. In contrast, Oh et al. [5] suggested that high debt may dissuade firms from committing to CSR, implying that the debt-to-assets ratio will harm CSR.

#### 3.3.2. Dividend payouts

We also follow Jiraporn and Chintrakarn [6] and Arora and Dharwadkar [12] to control dividend payouts (DIV), which are calculated by dividing the annual dividends by the total assets. Arora and Dharwadkar [12] argue that CSR may indicate agency losses in certain situations, so it is essential to account for any cash dividends paid out before detecting these losses. Lakhal et al. [40] found that dividend payout is positively associated with CSR engagement.

#### 3.3.3. Profitability

We follow Chen et al. [4] in controlling for profitability (ROA), which is calculated by dividing net income by total assets. They contended that organizations with greater ROA are more inclined to invest in CSR because they have more resources.

### 3.4. Model construction

To test for the agency theory, overinvestment hypothesis, career concern hypothesis, and career horizon hypothesis, we follow Jiraporn and Chintrakarn [6] to examine how CEO power (ADJCEO) affects CSR and add the non-linear variable ADJCEO*ADJCEO to examine whether there is a non-linear relationship between them. ADJCEO represents the index of CEO power with dimensions including CEO age, tenure, and ownership. Our baseline regression model is as follows:

**Model 1:**

$${\mathrm{CSR}}_{i,t}=\beta_{0}+\beta_{1}ADJCEO_{i,t-1}+\sum \beta_{q}control_{i,t-1}+\alpha_{i}+\alpha_{t}+\mu_{\mathrm{it}}$$ (1)

**Model 2:**

$${\mathrm{CSR}}_{i,t}=\beta_{0}+\beta_{1}ADJCEO_{i,t-1}+\beta_{2}ADJCEO_{i,t-1}*ADJCEO_{i,t-1}+\sum \beta_{q}{\mathrm{control}}_{i,t-1}+\alpha_{i}+\alpha_{t}+\mu_{\mathrm{it}}$$ (2)

To test for stakeholder theory and conflict-resolution hypothesis, we follow Jo and Harjoto [10] and Xiang et al. [11] to examine how investor attention (ADJIA) affects CSR. ADJIA represents the investor attention index using SVI.

**Model 3:**

$${\mathrm{CSR}}_{i,t}=\beta_{0}+\beta_{1}ADJIA_{i,t-1}+\sum \beta_{q}control_{i,t-1}+\alpha_{i}+\alpha_{t}+\mu_{\mathrm{it}}$$ (3)

In model (4), we integrate CEO power (ADJCEO) and investor attention (ADJIA) to explore the collective impact of these two variables on CSR, considering their simultaneous presence in the same model. In addition, our control factors, including LEV, DIV, and ROA, have varied effects on CSR [4–6, 8]. As a result, we investigate how control factors impact CSR across all models.

**Model 4:**

$${\mathrm{CSR}}_{i,t}=\beta_{0}+\beta_{1}ADJCEO_{i,t-1}+\beta_{2}ADJIA_{i,t-1}+\sum \beta_{q}control_{i,t-1}+\alpha_{i}+\alpha_{t}+\mu_{\mathrm{it}}$$ (4)

To test the stakeholder theory and conflict-resolution hypothesis, we include the interaction variable ADJCEO*ADJIA in the regression model, following the arguments of Jo and Harjoto [10], Xiang et al. [11], Arora and Dharwadkar [12], Duong et al. [16] This enables us to examine how investor attention moderates the relationship between powerful CEOs and CSR in family companies in Vietnam. The models are expressed as follows:

**Model 5:**

$${\mathrm{CSR}}_{i,t}=\beta_{0}+\beta_{1}ADJCEO_{i,t-1}+\beta_{2}ADJIA_{i,t-1}+\beta_{3}ADJCEO_{i,t-1}*ADJIA_{i,t-1}+\sum \beta_{q}control_{i,t-1}+\alpha_{i}+\alpha_{t}+\mu_{\mathrm{it}}$$ (5)

Where "i" is cross-sections, "t" is time, and "α" is the intercept. Besides, CSR represents the corporate social responsibility index; ADJCEO is the CEO power index adjusted by industry; ADJIA represents investor attention adjusted by industry; Control includes the leverage ratio of business (LEV), business dividend payouts ratio (DIV) and profitability (ROA). α_i_ is the firm fixed effect, and α_t_ is the year fixed effect. μ_it_ is the residual value. S2 Appendix contains all variable definitions.

### 3.5. Estimation procedure

This study initially conducted the Hausman and Redundant Tests to select the optimal estimation approach among Pooled Ordinary Least Squares (OLS), Fixed Effects Model (FEM), and Random Effects Model (REM). However, these methods were found to violate assumptions related to autocorrelation, heterogeneity, and endogeneity [41]. Subsequently, following the methodology outlined by Duong et al. [13], this study utilized Durbin-Watson, Wald, and Durbin-Wu-Hausman tests to identify such violations. The results confirm their presence.

Therefore, this study follows Almustafa et al. [14], Duong et al. [42] and Vo et al. [15] to implement the two-step system generalized method of moments (GMM) regression and perform specification and validation tests to ensure its compatibility and efficiency in our empirical analyses. The two-step system GMM method enables us to categorize all explanatory variables and equations regarding differences and levels [14]. In addition, it additionally mitigates biases resulting from potential endogeneity, such as unobserved heterogeneity, omitted variable bias, and measurement error [15]. Notably, the GMM technique offers ease of application and resilience in heteroskedasticity while making no assumptions regarding cross-equation error correlation or homoskedasticity [43]. Finally, the two-step dynamic System Generalized Method of Moments (Sys-GMM) was employed by the approach advocated by Chi et al. [44], Almustafa et al. [14], Vo et al. [15], which has been demonstrated to outperform the Dif-GMM.

In addition, the suitability of three statistical tests, including the F-test and J-statistic, are also evaluated. The Fisher test assesses the significance of the estimated coefficients, and the J-statistic is utilized to identify endogeneity. The estimated coefficients are considered statistically significant if the probability value of the Fisher test is less than 1%. Furthermore, if the probability value of the J-statistic is higher than 20%, it indicates no endogeneity issues in the model. Finally, the study employs the approach outlined in Duong et al. [16] to conduct a robustness examination to verify the longevity and consistency of our key empirical outcomes in different subgroups categorized by the age of the CEO.

## 4. Empirical results

### 4.1. Descriptive statistics

Table 1 presents the descriptive statistics of the samples. The CSR mean value is 2.1665, and its standard deviation is about 1.3092, which aligns with Duong et al. [16]. Furthermore, it signifies attaining a score of 2.1665 out of a maximum benchmarked composite CSR information score of 4.00 within the Vietnamese family businesses context. ADJCEO has an average value of -0.1380, consistent with studies of Altunbaş et al. [19] and Brodmann et al. [20] from the US. In addition, Vietnam’s CEO power is lower than that of the US, with both their results for PCA of CEO power having means of 0.00 and 0.438. Moreover, the average ADJCEO of -0.1380 indicated that most family businesses have CEOs’ power lower than the industry median. The ADJIA average value is approximately -0.0232, with a standard deviation of 0.6774. This result also means that most family businesses have less investor attention than the industry median. Furthermore, Table 1 reports that the LEV’s mean value is 0.5007 (50.07%), and its standard deviation is 0.1792. Additionally, Table 1 provides descriptive statistics for other control variables, such as LEV, DIV, and ROA, representing the companies’ leverage, dividend payout ratio, and profitability. The average debt-to-asset ratio is 0.5007 (50.07%). The result is consistent with Na et al. [9] and Rauf et al. [29] in the context of China businesses’ debt-to-asset ratio, at around 0.50. This result represents that family businesses in Vietnam rely moderately on debt financing to support their operations and investments. A ratio of around 50% indicates a relatively healthy balance between debt and assets. Finally, the dividend payout ratio (DIV) and return on asset (ROA) have average values of 0.0003 and 0.0565, respectively.

**Table 1:** Descriptive statistics of variables.

	Obs	Mean	Median	95^th^ Pct	5^th^ Pct	Std. Dev.
CSR	985	2.1665	2	4	0	1.3092
ADJCEO	985	-0.1380	-1.1276	34.0905	-32.5074	19.1174
ADJIA	985	-0.0232	0.1048	0.8684	-1.0000	0.6774
LEV	985	0.5007	0.5200	0.7600	0.2198	0.1792
DIV	985	0.0003	0.0002	0.0010	0.0000	0.0004
ROA	985	0.0565	0.0454	0.1443	0.0023	0.0467

Note: Table 1 represents descriptive statistics. From 2005 through 2020, the sample data comprises 985 yearly observations from 116 family enterprises in Vietnam. S2 Appendix contains all variable definitions.

### 4.2. Pearson correlation matrix

Table 2 presents the research data sample by the correlation matrix. Correlation analysis is employed to ascertain the presence of multicollinearity [29]. According to Na et al. [9], strong multicollinearity is indicated when the correlation coefficient within the correlation matrix of independent variables exceeds 0.80. The correlation coefficient in our analysis is between 0.01 and 0.33. Therefore, the correlation relationships between the independent variables are moderate. Moreover, this study examined the variance inflation factor (VIF) to ensure that no multicollinearity issue was present in our sample. According to the result, the study does not suffer from multicollinearity issues [9, 29, 45].

**Table 2:** Pearson correlation matrix.

	ADJCEO	ADJIA	LEV	DIV	ROA	VIF
ADJCEO	1					1.00329
	-----					
ADJIA	-0.03518	1				1.00868
	(0.2700)	-----				
LEV	0.02203	-0.07062**	1			1.13281
	(0.4899)	(0.0267)	-----			
DIV	0.04061	-0.04615	-0.04772	1		1.00770
	(0.2028)	(0.1478)	(0.1345)	-----		
ROA	-0.01516	0.01322	-0.33066***	-0.01807	1	1.12444
	(0.6346)	(0.6785)	(<0.001)	(0.5710)	-----	

Note: Table 2 provides the Pearson correlation matrix. From 2005 through 2020, the sample data comprises 985 yearly observations from 116 Vietnamese family companies. S2 Appendix contains a list of all variable definitions. The symbols ***, **, and * indicate statistical significance levels of 1%, 5%, and 10%, respectively.

### 4.3. How CEO power and investor attention affect CSR

Table 3 shows insignificant relationships between CEO power, investor attention, and CSR, except for the inverted U-shape relationship between CEO power and CSR. The result from the Durbin-Watson and Wald tests shows that all five models have autocorrelation and heteroskedasticity. In addition, the F-test shows that the estimated coefficients are statistically significant, except for the results from Models 1 and 3.

**Table 3:** Regressions result from REM and FEM methods.

Variable	Model 1	Model 2	Model 3	Model 4	Model 5
	REM	FEM	REM	REM	FEM
ADJCEO	0.0025	0.0029*		0.00254	0.0021
	(0.1312)	(0.0943)		(0.1303)	(0.2238)
ADJCEO*ADJCEO		-0.0005***			
		(<0.001)			
ADJIA			0.0320	0.03187	0.0339
			(0.7033)	(0.7055)	(0.6109)
ADJCEO*ADJIA					-0.0009
					(0.7043)
LEV	-0.0586	-0.2772	-0.0500	0.03187	-0.1083
	(0.8895)	(0.4224)	(0.9060)	(0.8919)	(0.7593)
DIV	214.8845	94.5094	219.0875	214.8822	162.2323
	(0.1186)	(0.3711)	(0.1105)	(0.1188)	(0.1325)
ROA	-1.0360	-2.2952	-1.0876	-1.06270	-1.7682*
	(0.4431)	(0.0205)	(0.4225)	(0.4315)	(0.0812)
C	2.2845***	2.6021***	2.2819***	2.28615*	2.2691***
	(<0.001)	(<0.001)	(<0.001)	(<0.001)	(<0.001)
Firm fixed effect	Yes	Yes	Yes	Yes	Yes
Period fixed effect	No	Yes	No	No	Yes
Number of obs	985	985	985	985	985
R-squared	0.0088	0.5229	0.0069	0.00908	0.4992
Adjusted R-squared	0.0048	0.4567	0.0029	0.00402	0.4290
F-statistic	2.1800	7.8926	1.7120	1.7942	7.1095
Prob(F-statistic)	0.0693	<0.001	0.1451	0.1113	<0.001
Prob (Redundant Test)	<0.001	<0.001	<0.001	<0.001	<0.001
Prob (Hausman Test)	0.1472	0.0745	0.3246	0.9480	0.0436
Prob (Wald test)	<0.001	<0.001	<0.001	<0.001	<0.001
Hannan-Quinn criteria.		3.1099			3.1624
Durbin-Watson stat	1.8250	1.6326	1.8251	1.8274	1.9433

Note: Table 3 illustrates the FEM and REM estimates. From 2005 through 2020, the sample data comprises 985 yearly observations from 116 Vietnamese family companies. S2 Appendix contains all variable definitions. The symbols ***, **, and * indicate statistical significance levels of 1%, 5%, and 10%, respectively.

After employing FEM and REM, the study employs the Durbin-Wu-Hausman test to determine if endogenous variables exist. To begin with, the sample for the ADJCEO endogenous examination test model is as follows:

**Model 6:**

$${\mathrm{ADJCEO}}_{i,t}=\beta_{0}+\beta_{1}ADJIA_{i,t}+\beta_{2}LEV_{i,t}+\beta_{3}DIV_{i,t}+\beta_{4}ROA_{i,t}+\mu_{\mathrm{it}}$$ (6)

Model 4 incorporates the residuals of ADJCEO (Res-ADJCEO) obtained in the first stage as an extra variable of ADJCEO [42]. When the coefficient of the residual is statistically significant, it indicates the presence of an endogeneity problem. Similarly, we examine additional variables like ADJIA, LEV, DIV, and ROA.

According to Table 4, ADJCEO and DIV are endogenous variables since their residual coefficients are statistically significant. As a result, we apply the dynamic system GMM to eliminate estimation bias.

**Table 4:** Coefficient of residual variables.

Residual	Coefficient	P-value
Res-ADJCEO	0.00281*	0.0729
Res-ADJIA	-0.036	0.546
Res-LEV	-0.10374	0.7452
Res-DIV	6.12388**	0.0353
Res-ROA	0.858867	0.3719

Note: Table 4 represents the results of the Durbin-Wu-Hausman test. The symbols ***, **, and * reflect the significance levels of 1%, 5%, and 10%, respectively.

Table 5 shows the regression results of the GMM method. According to Table 5, the P-value of the J-statistic is above 20%, which means that all instrument variables are valid, and the models have no endogeneity issues.

**Table 5:** Regressions result from the GMM method.

Variable	Model 1	Model 2	Model 3	Model 4	Model 5
Lag of Dep. Var	0.2488***	0.2193***	0.2429***	0.2458***	0.2578***
	(<0.0001)	(<0.0001)	(<0.0001)	(<0.0001)	(<0.0001)
ADJCEO	0.0027***	0.0036***		0.0029***	0.0028***
	(<0.0001)	(<0.0001)		(<0.0001)	(0.0003)
ADJCEO*ADJCEO		-0.0003***			
		(<0.0001)			
ADJIA			-0.1443***	-0.1429***	-0.1573***
			(<0.0001)	(<0.0001)	(<0.0001)
ADJCEO*ADJIA					0.0053***
					(<0.0001)
LEV	1.0219***	1.0502***	1.0131***	1.0581***	1.3257***
	(<0.0001)	(<0.0001)	(<0.0001)	(<0.0001)	(<0.0001)
DIV	1.0728***	2.3928***	1.1269***	1.0032***	1.4488***
	(<0.0001)	(0.0090)	(<0.0001)	(<0.0001)	(0.0012)
ROA	-0.3359***	-0.0374	-0.0834**	-0.1419	0.2786
	(<0.0001)	(0.5458)	(0.0298)	(0.1762)	(0.5337)
Cross-section fixed (first differences)					
Turning point		6.06250			
Number of obs	761	761	761	761	761
J-statistic	75.5936	72.8737	74.4661	75.8129	68.6208
Prob(J-statistic)	0.3947	0.4491	0.4303	0.35657	0.5580

Note: Table 5 displays the GMM estimate results. From 2005 through 2020, the sample data comprises 985 yearly observations from 116 family enterprises in Vietnam. S2 Appendix contains a list of all variable definitions. The symbols ***, **, and * reflect the statistical significance levels of 1%, 5%, and 10%, respectively.

Finally, we follow Duong et al. [16] to conduct a robustness test to confirm the durability of our significant findings across subsamples by CEO age. The findings are reported in Table 6.

**Table 6:** Robustness test results from GMM regression.

Variable	OVER 30		OVER40		OVER50		
	Model 2	Model 5	Model 2	Model 5	Model 2	Model 5	
Lag of Dep. Var	0.1791***	0.2262***	0.0689***	0.0985***	0.1544***	0.1710***	
	(<0.0001)	(<0.0001)	(<0.0001)	(<0.0001)	(<0.0001)	(<0.0001)	
ADJCEO	0.0045***	0.0043***	0.0042***	0.0011**	0.0119***	0.0065***	
	(<0.0001)	(<0.0001)	(<0.0001)	(0.0234)	(<0.0001)	(<0.0001)	
ADJCEO*ADJCEO	-0.0003***		-0.0003***		-0.0005***		
	(<0.0001)		(<0.0001)		(<0.0001)		
ADJIA		-0.176***		-0.2243***		-0.0058	
		(<0.0001)		(<0.0001)		0.9238	
ADJCEO*ADJIA		0.0026***		0.0041***		-0.0073***	
		(<0.0001)		(<0.0001)		(0.0005)	
LEV	1.3496***	1.6053***	1.8677***	2.2217***	-0.3025***	-0.1095	
	(<0.0001)	(<0.0001)	(<0.0001)	(<0.0001)	0.0117	(0.8257)	
DIV	-5.5586	80.219***	26.355***	139.57***	-523.82***	-357.31***	
	(0.6927)	(<0.0001)	(0.0676)	(<0.0001)	(<0.0001)	(0.0001)	
ROA	-1.2740***	-1.282***	-2.3066***	-2.3230***	-5.6571***	-4.6861***	
	(<0.0001)	(<0.0001)	(<0.0001)	(<0.0001)	(<0.0001)	(<0.0001)	
Cross-section fixed (first differences)						
Turning point	7.50		7.00		11.90		
Number of obs	736	736	605	605	319	319	
J-statistic	77.408	66.103	63.950	66.434	46.640	39.147	
Prob(J-statistic)	0.3103	0.6423	0.6167	0.4966	0.3251	0.5969	

Note: Table 6 shows robustness test results using GMM regressions on a sample of 116 family firms in Vietnam from 2005 to 2020, with CEOs aged 30–50. S2 Appendix contains a list of all variable definitions. The symbols ***, **, and * signify the significant levels of 1%, 5%, and 10%, respectively.

## 5. Discussion

**Fig 1:**
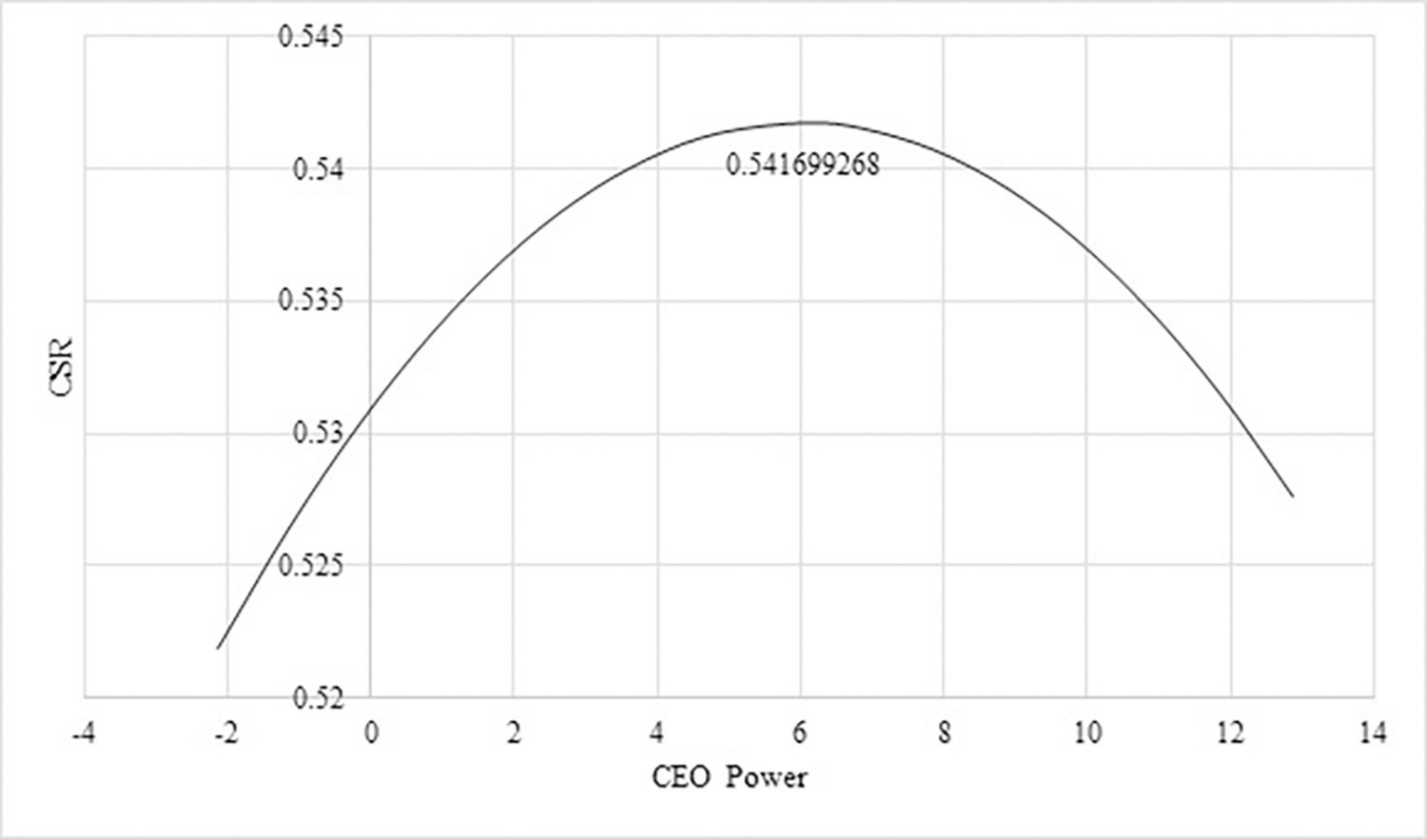
CEO overpower and CSR (source: author’s calculation).

Table 5 highlights exciting results on how CEO power (ADJCEO) affects Vietnamese-listed family businesses’ CSR engagements. The findings show a significant inversed U-shape relationship between CEO power and CSR in Model 2. These results indicate that powerful CEOs initially positively impact CSR engagements; one unit increase in CEO power enhances CSR by 0.0036 units. However, when CEO power surpasses a certain threshold, a unit increase in CEO power leads to a decline in CSR engagements of -0.0003 units. This study further follows Jiraporn and Chintrakarn [6] to estimate the turning point of CEO power by taking the absolute value of $\frac{{\beta 1}_{ADJCEO}}{2*({\beta 2}_{{ADJCEO}^{2}})}$ in Model 2. Based on the estimated coefficients of Model 2, with a calculated threshold of CEO power at 6.06250 units, CSR engagements peaked at approximately 0.542 units. This result indicates that maximizing the enhancement of CEO power fosters CSR up to a level of 0.542 units. However, beyond this threshold, further empowerment of CEOs in family-owned businesses diminishes CSR initiatives. Accordingly, the relationship between CEO power and CSR engagement is presented in Fig 1.

The result of CEO power and its effect on CSR aligns with the findings of Jiraporn and Chintrakarn [6], suggesting that the CEO may profit personally from CSR initiatives. However, as CEOs gain more power and become more secure, they may reduce CSR investment. This finding is because they perceive CSR as a non-essential expense that hinders their ability to maximize free cash flow and allocate resources toward other profit-generating activities. This finding also supports the overinvestment hypothesis and implies that managers and insiders are motivated to overinvest in CSR when such investments yield private benefits like reputation enhancement. Since CEO tenure is one of our proxies, our finding is consistent with Chen et al. [4]. They stated that CEOs are motivated to prioritize CSR during the early stages of their leadership to mitigate potential career concerns. This result is because CSR performance is a crucial component of their performance evaluation, and strong CSR performance at the beginning of their tenure is linked to a lower likelihood of being replaced as CEO (career concern hypothesis). Moreover, CEOs in the initial stages of their tenure exhibit more significant incentives to invest in CSR than those in later stages because CSR initiatives can offer long-term advantages for the company, which can benefit the CEO later in their career Chen et al. [4]. In terms of CEO age, Oh et al. [5] argued that CEOs who are approaching or have exceeded the typical retirement age may have less incentive to engage in CSR due to the long-term nature of its benefits. Consequently, they may be less motivated to make socially responsible decisions (career horizon hypothesis). On the other hand, younger CEOs with longer career horizons may be more interested in investing in CSR because they stand to reap greater rewards from such engagement. Moreover, it aligns with the results and arguments of Chen et al. [4], Oh et al. [5], and Jiraporn and Chintrakarn [6]. The results support hypothesis 3, agency theory, overinvestment hypothesis, career concern hypothesis, and career horizon hypothesis. Our study reveals a previously seldom unrecognized non-linear relationship, diverging from conventional perspectives. Therefore, this finding contributes to the existing corporate governance literature and enriches our understanding beyond the prevailing linear paradigms.

The result reports an inversed U-shape relationship between CEOs’ power and CSR in the context of Vietnamese family businesses. This finding does not support the linear relationship between these two variables and the stakeholder theory, which posits that CEOs engage in CSR activities to increase the firm value and balance stakeholders’ interests rather than seek personal reputation enhancement or private benefits [3]. In Vietnamese family businesses, CEOs may be motivated by personal reputation enhancement, networking opportunities, control, and financial incentives, which can lead them to prioritize their interests over those of the firm and its stakeholders. In Vietnamese society, personal reputation and social status hold significant importance. Engaging in CSR activities can enhance the CEO’s reputation within the community among business associates. In addition, due to higher reputations as socially responsible individuals, participation in CSR initiatives provides opportunities for CEOs to network with other influential individuals, government officials, and business leaders.

Table 5 shows a significant inverse association between investor interest and CSR involvement. In particular, a one-point rise in investor attention reduces the CSR index by 0.1573 points, based on the coefficient of Model 5. If CG mechanisms prioritize short-term results, the expenses linked with CSR may surpass the benefits. Furthermore, according to Arora and Dharwadkar [12], institutional investors who focus on reaching short-term objectives and performance targets may not encourage managers to participate in CSR initiatives because of the unpredictability of outcomes. Therefore, our research validates hypothesis 5 and the conflict-resolution hypothesis, which suggests an inverse relationship exists between investor attention and CSR engagement. Prior studies have largely remained at the level of theoretical arguments or provided results that imply the relationship indirectly, as exemplified by Arora and Dharwadkar [12], Radu and Smaili [21], and Bhuiyan and Nguyen [22]. This research provides novel insights that clarify the relationship between investor attention and CSR.

Regarding investor attention, the result is inconsistent with the stakeholder theory. In Vietnamese family businesses, investor attention negatively affects CSR engagement primarily due to a short-term focus, risk aversion, and resource allocation priorities. Specifically, institutional investors, particularly those with short-term objectives and performance targets, may prioritize immediate financial returns over long-term sustainability. In addition, institutional investors may perceive CSR activities as risky investments with uncertain outcomes. This risk aversion and the unpredictability of CSR outcomes can lead investors to prefer safer, more predictable investment opportunities that yield quicker returns. Consequently, they may not advocate for CSR initiatives, as they perceive them as potentially jeopardizing short-term financial performance. Finally, in the face of limited resources, particularly in smaller family businesses, increased investor attention may lead managers to prioritize investments directly contributing to short-term financial performance.

Table 5 reveals that CEO power functions as a moderator in the relationship between investor attention and CSR. Specifically, based on Model 5, powerful CEOs in firms with high investor attention exhibit a 0.53 percentage point increase in the CSR index compared to other companies. This finding aligns with the agency theory and overinvestment hypothesis, which suggest that CEO power can lead to excessive investments in CSR [3]. Managers could be driven to engage in excessive CSR investments to attain personal advantages, including establishing a favorable reputation as socially responsible individuals [10]. They also stated that increased investor attention should reduce the incentive and opportunities for overconfident CEOs and insiders to overinvest in CSR initiatives to build their reputations because institutional investors who prioritize meeting short-term objectives and performance targets may not advocate for managers to participate in CSR initiatives [12]. However, our findings show that powerful CEOs in family firms in Vietnam would overinvest in CSR owing to agency theory and overinvestment hypothesis, given that their companies receive great investor attention. CEOs with greater control can make decisions disregarding investor interests [9]. Although this finding contradicts certain prior studies and arguments, it aligns with the propositions put forth by Sheikh [3], Jo and Harjoto [10], Na et al. [9], hypothesis 6, the agency theory, and the overinvestment hypothesis.

According to the findings in Table 5, there is a positive correlation between leverage (LEV) and dividend payouts (DIV) with CSR. As per the results from Model 5, a 1% rise in leverage corresponds to 1.3257 points increase in CSR, while a 1% increase in dividend payout is associated with 1.4488 points rise in CSR. Our results are consistent with Chen et al. [4], Jiraporn and Chintrakarn [6], Arora and Dharwadkar [12], and Lakhal et al. [40]. According to Chen et al. [4], enterprises with idle financial resources are more able to afford CSR. It is necessary to account for any cash dividends paid out before detecting agency losses from CSR investments [12]. In addition, ROA negatively affects CSR but is not significant in most models.

Table 6 demonstrates the robustness test from GMM regression for the significant results. The results are robust and provide exciting results related to how the moderating role of CEO power on the relationship between investor attention and CSR significantly changed through different CEO ages. Specifically, the inverted U-shape relationship between CEO power and CSR remains unchanged. In addition, the main findings of the moderating role are also robust across subsamples of CEOs over 30 and 40. However, powerful CEOs over 50 in family businesses and under high investor attention hurt CSR. Due to goal conflicts linked to time horizons and uncertainty of outcomes, close investor attention should diminish the motivation and possibilities for overconfident CEOs and insiders to overinvest in CSR [10].

## 6. Conclusion

This research centers on the impact of CEO power and investor attention on corporate social responsibility (CSR) initiatives in businesses. Furthermore, it investigates whether CEO power moderates the relationship between investor attention and CSR engagement, specifically within family businesses. Our sample is an unbalanced panel with 985 yearly observations from 2005 to 2020. This study employs the GMM method to address autocorrelation, endogeneity and heteroskedasticity issues. The study provides interesting results. Firstly, a notable inverse U-shaped relationship exists between the CEO power level and the degree of engagement in CSR. Secondly, heightened investor attention correlates with a reduction in CSR initiatives. Finally, CEOs’ power significantly moderates the association between investor attention and CSR. Nevertheless, once CEOs surpass the age of 50, the impact of CEO power on CSR endeavors is moderated by investor attention. The results support the agency theory, career concern hypothesis, care horizon hypothesis, and conflict-resolution hypothesis.

**Fig 2:**
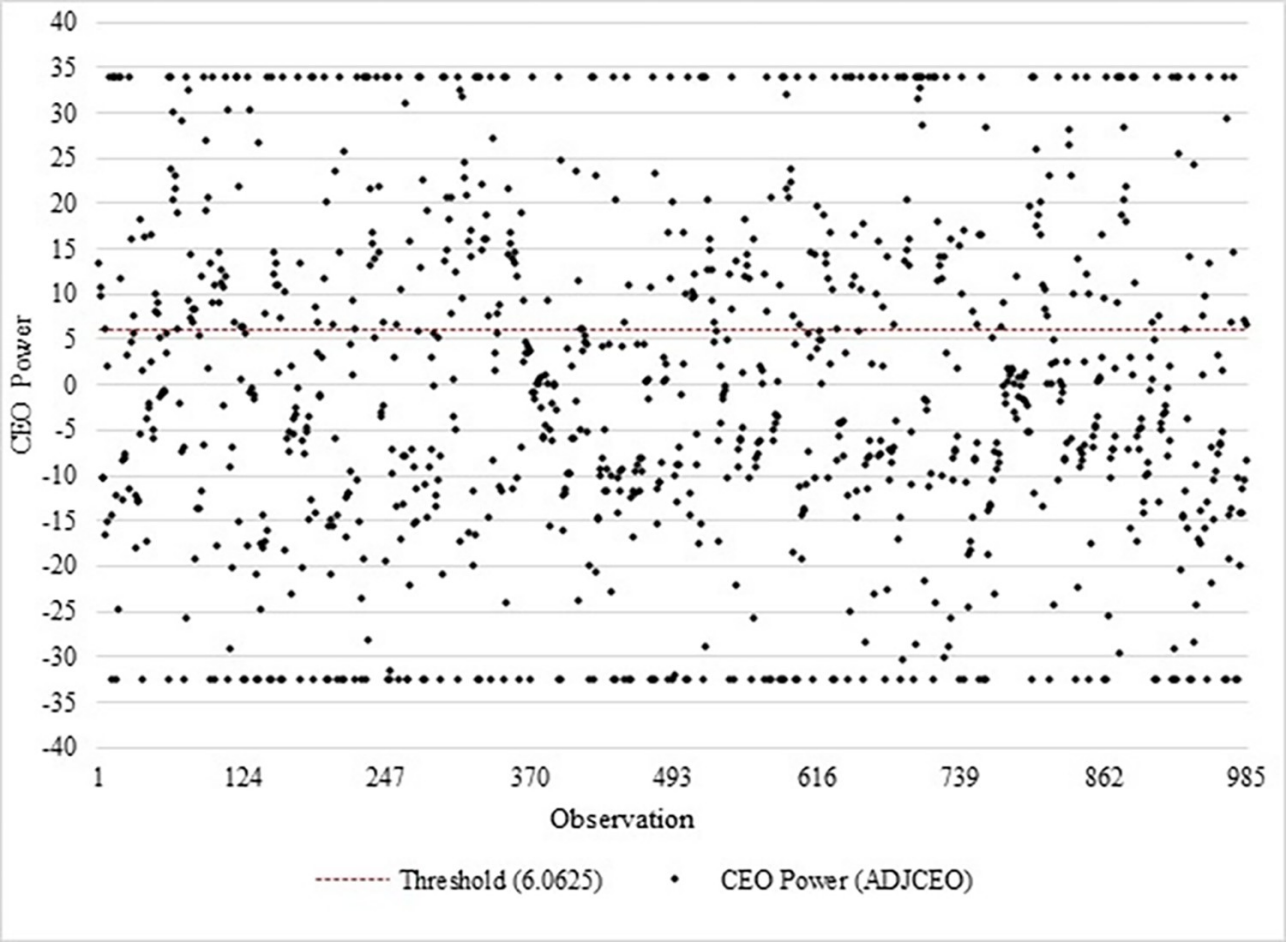
CEO power observations and its threshold (source: author’s calculation).

This research offers practical insights and implications for managers and investors in family businesses seeking to improve their long-term performance in emerging markets. With increased investor scrutiny, companies may be compelled to improve their accountability and transparency regarding CSR activities. This can lead to better reporting practices, clearer stakeholder communication, and a stronger commitment to CSR goals. Stakeholders should advocate for accountable governance structures and uphold ethical leadership to ensure CSR investments benefit society and align with stakeholders’ interests. In addition, unchecked CEO power may lead to an excessive allocation of resources towards CSR due to the agency theory and career concern hypothesis, potentially compromising the firm’s financial well-being. Therefore, establishing robust mechanisms for transparency and oversight is essential to monitor CEO conduct effectively. As Zahid et al. [46] emphasized, enhancing organizational performance necessitates considering executive characteristics as drivers of CSR reporting. Policies should be formulated to mitigate executive turnover and influence while prioritizing appointing executives with desirable attributes, such as Chief Financial Officers (CFOs). Encouraging the appointment of executives with specific attributes, such as CFOs who may contribute positively to CSR reporting, could be a strategic move. Additionally, managing the power of CEOs is highly crucial; investors should pay close attention to the complex dynamics of CEO power within these firms, especially its influence on Corporate Social Responsibility (CSR) efforts. Specifically, as depicted in Fig 2, about 35.94% of total observations show CEO power exceeding the estimated optimal value (6.0625), while 65.06% fall below it. This indicates that companies in the latter category might benefit from increasing CEO power to enhance CSR impact. In contrast, those in the first category should consider reducing CEO power to mitigate its adverse effects on CSR engagement. Moreover, collaborative efforts among stakeholders are vital for promoting sustainable performance and instilling responsible governance practices within family businesses, fostering positive social impact and enduring financial resilience in emerging markets.

This study contributes significantly to the expanding literature on corporate governance within the domain of family businesses. Nevertheless, it is imperative to acknowledge certain inherent limitations in our research. Firstly, our dataset is confined to the specific context of Vietnam, which may hinder the generalizability of our findings to other geographical regions or nations. Additionally, considering Vietnam’s status as a frontier market, the dynamics observed in our study may diverge from those prevalent in more mature or developed economies. Furthermore, specific CEO power indicators have yet to be incorporated into the analyses due to data availability limitations. Specifically, this study has yet to explore variables such as CEO duality and CEO compensation. For future research lines, it is recommended that future research endeavors broaden the scope of data collection and undertake comparative analyses across diverse countries. This approach will facilitate a more comprehensive understanding of the mechanisms through which CEO power and investor attention impact corporate social responsibility within both developed and emerging markets. Cross-national comparisons are pivotal for garnering nuanced insights and fostering a deeper comprehension of the intricate interplays. Ultimately, these concerted efforts will engender more robust and insightful outcomes, advancing scholarly discourse in corporate governance and family businesses.

## Supporting information

S1 Appendix (DOCX)

S2 Appendix (DOCX)
