# Supplementary material for: Investor attention and corporate social responsibility of family businesses in Vietnam: The moderating role of CEO overpower
Source: PLoS One. 2024 Jul 19;19(7):e0306989. doi: 10.1371/journal.pone.0306989 (PMC11259297; doi:10.1371/journal.pone.0306989)
Supplement: S1 Appendix — (DOCX) [file pone.0306989.s001.docx]

| **Appendix A**  The result of the PCA for CEO overpowers. | | | | | |
| --- | --- | --- | --- | --- | --- |
| Eigenvalues of matrix | | | | | |
| (a) Eigenvalues | | | | | |
| Number | Value | Difference | Proportion | Cumulative |  |
| 1 | 1.461387 | 0.596173 | 0.4871 | 0.4871 |  |
| 2 | 0.865214 | 0.191815 | 0.2884 | 0.7755 |  |
| 3 | 0.673399 | --- | 0.2245 | 1.0000 |  |
| (b) Eigenvectors (loadings): | | | | | |
| Variable | PC 1 | PC 2 | PC 3 |  |  |
| CEO_AGE | 0.583962 | -0.549356 | 0.597659 |  |  |
| CEO_TENURE | 0.640009 | -0.141338 | -0.755256 |  |  |
| CEO_OWN | 0.499377 | 0.823548 | 0.269057 |  |  |
