# Supplementary material for: Investor attention and corporate social responsibility of family businesses in Vietnam: The moderating role of CEO overpower
Source: PLoS One. 2024 Jul 19;19(7):e0306989. doi: 10.1371/journal.pone.0306989 (PMC11259297; doi:10.1371/journal.pone.0306989)
Supplement: S2 Appendix — (DOCX) [file pone.0306989.s002.docx]

| **Appendix B**  Variables descriptions. | | | |  |
| --- | --- | --- | --- | --- |
| Variable | Notation | Variable descriptions | Reference | |
| Dependent variables |  |  |  |  |
| Corporate social responsibility | CSR | CSR is rated on a 4-point scale with four criteria: ENV, COM, EMW, and PCS. | Duong et al. [16], Aggarwal and Singh [36] | |
| Independent variables |  |  |  |  |
| CEO overpower | ADJCEO | The adjusted CEO overpower index is measured by three criteria: CEO age, CEO tenure, and CEO ownership. | Duong et al. [16], Altunbaş et al. [19], Brodmann et al. [20] | |
| Investor attention | ADJIA | Adjusted aggregate search frequency in Google Search Volume Index (SVI). | Da et al. [23] | |
| Control variables |  |  |  |  |
| Leverage ratio | LEV | Total liabilities to total assets. | Chen et al. [4] | |
| Payout ratio | DIV | Yearly dividend to total assets | Arora and Dharwadkar [12] | |
| Profitability | ROA | Net income to total assets | Jiraporn and Chintrakarn [6] | |
